# Effect of D-Mannitol on the Microstructure and Rheology of Non-Aqueous Carbopol Microgels

**DOI:** 10.3390/ma14071782

**Published:** 2021-04-04

**Authors:** Simona Migliozzi, Panagiota Angeli, Luca Mazzei

**Affiliations:** Department of Chemical Engineering, University College London, Torrington Place, London WC1E 7JE, UK; simona.migliozzi.16@ucl.ac.uk

**Keywords:** carbopol, microgels, nonlinear rheology, LAOS, yield stress, structure-rheology relationship, mannitol, fluorescence microscopy, non-aqueous formulations

## Abstract

D-mannitol is a common polyol that is used as additive in pharmaceutical and personal care product formulations. We investigated its effect on the microstructure and rheology of novel non-aqueous Carbopol dispersions employing traditional and time-resolved rheological analysis. We considered two types of sample, (i) fresh (i.e., mannitol completely dissolved in solution) and aged (i.e., visible in crystalline form). The analysis of the intracycle rheological transitions that were observed for different samples revealed that, when completely dissolved in solution, mannitol does not alter the rheological behaviour of the Carbopol dispersions. This highlights that the chemical similarity of the additive with the molecules of the surrounding solvent allows preserving the swollen dimension and interparticle interactions of the Carbopol molecules. Conversely, when crystals are present, a hierarchical structure forms, consisting of a small dispersed phase (Carbopol) agglomerated around a big dispersed phase (crystals). In keeping with this microstructural picture, as the concentration of Carbopol reduces, the local dynamics of the crystals gradually start to control the integrity of the microstructure. Rheologically, this results in a higher elasticity of the suspensions at infinitesimal deformations, but a fragile yielding process at intermediate strains.

## 1. Introduction

Formulated products with complex textures are part of everyday life; examples include cosmetic and healthcare products, drug delivery devices, building materials, food, and inks. These materials typically comprise multiple phases and active agents, which must be carefully balanced and tailored to ensure the desired functionality and texture of the product. In this context, microgel suspensions are a primary class of materials, extensively employed in the design of novel formulations because of their unique colloid-polymer duality [1] and the chemical design flexibility that they offer [2,3,4]. In general, microgels are identified as soft colloidal particles that consist of polymer networks that swell when dispersed in a solvent. When microgels are used as rheology modifiers, knowing how different solvents and active ingredients affect the swelling and interparticle interactions of the microgel particles is fundamental to ensure the efficient rheological design of a product.

Among the commercially available molecules, Carbopol polymers are one of the most popular microgels employed industrially and in research. These molecules consist of high molecular weight polymers of polyacrylic acids, which swell upon dispersion in a polar solvent, creating a transparent suspension of soft elastic particles. Thanks to their nontoxicity, stability, high thickening properties, and transparency, Carbopol gels have been widely used in cosmetics, pharmaceuticals [5,6,7], and other commercial and industrial applications [8], as well as model yield stress fluids in academia [9,10,11]. In the majority of the applications, Carbopol dispersions are prepared in an aqueous environment. Owing to the high crosslinking density, Carbopol is insoluble in de-ionized water, i.e., in the absence of an osmotic gradient, water does not diffuse into the polymeric network. Hence, to induce swelling, one needs to add a base (e.g., sodium hydroxide, triethanolamine) to the aqueous solution during the preparation to neutralize the acidic groups and cause the ionization of the carboxylate groups. Similar to most polyelectrolytes, the final swollen Carbopol configuration depends on the internal density of uncompensated ions on the polymer backbone and, therefore, can be controlled through the pH of the solution [12]. For this reason, researchers have investigated extensively, for many types of Carbopol molecules and alkaline compounds, how changing the solution pH affects the rheological properties of water-based Carbopol microgels [13,14,15,16,17,18,19]. In addition to typical bases, the effect of other additives, such as surfactants and polyols, commonly used in the formulation of pharmaceutical and personal care products, has also been partly examined. Barreiro-Iglesias and co-workers [20,21] studied the effect of ionic and non-ionic surfactants on the viscosity of aqueous Carbopol gels. They found that the rheological properties of the microgels are strongly influenced by the type of surfactant, which controls the binding mechanism between surfactant molecules and Carbopol, thus influencing the final swelling of the particles and their interactions [22]. More recently, Lefrançois et al. [23] examined the effect of two polyols, mannitol and sorbitol, on the rheology and microstructure of neutralized aqueous Carbopol dispersions, revealing that a small concentration of mannitol can lead to a more heterogeneous network of Carbopol particles, a finding that they tentatively attributed to the ability of mannitol to induce hydrogen bonds within the microgel particles.

On the contrary, the swelling behaviour of Carbopol molecules in the absence of water and the effect of the addition of active molecules, when the ionic forces are weaker, is still poorly understood. The use of organic matrices is central in various therapeutical applications that require the dissolution of highly hydrophobic drugs or the transport of compounds that need in-situ activation with water, thus highlighting the importance of a thorough rheological investigation of non-aqueous Carbopol dispersions. Polar solvents, such as glycerol and poly(ethylene glycol) of low molecular weight, represent a good alternative to water-based solutions, since they present a better affinity with the Carbopol network compared to water and can initiate the swelling of Carbopol molecules in the absence of neutralizing agents [24]. Recently [25], we showed that the use of glycerol, poly(ethylene glycol), or a combination of the two solvents mainly impacts the final swollen dimension of Carbopol particles, without significantly changing the interparticle interactions. This entails that the rheology of these dispersions scales with the particle volume fraction, independently of the solvent used, showing a flow behaviour consistent with that of soft particle systems in the athermal regime [26,27,28], with some anomalies being observed at significantly high particle concentrations, probably related to the morphological irregularities (i.e., uneven crosslinking density, dangling ends) of Carbopol particles. How active compounds would impact the internal microstructure and, in turn, the rheological behaviour of these non-aqueous dispersions is still unexplored.

Hence, this work aims to explore the effect of a polyol additive, D-mannitol, which is commonly added to pharmaceutical and food formulations as a sweetener and humectant, on the microstructure and rheology of Carbopol non-aqueous dispersions. Using the same Carbopol system investigated previously (Carbopol 974P NF) [25], we study the rheology of dispersions of Carbopol in a mixture of glycerol and poly(ethylene glycol) (solvents ratio 70%/30% wt) (i) in the absence of mannitol and (ii) for two different mannitol concentrations. Note that the type of Carbopol used corresponds to the same grade that is used by Lefrançois et al. [23]; this allows us to compare the effect of D-mannitol on the microstructure of aqueous and non-aqueous Carbopol suspensions. To disentangle the roles of the two dispersed phases (i.e., Carbopol and mannitol), we first mapped the linear viscoelastic behaviour of the dispersions at increasing Carbopol concentrations and observed the onset of the liquid-to-solid transition for different amounts of mannitol dissolved in solution. Subsequently, we investigated the critical yielding behaviour of Carbopol dispersions at sample concentrations just above and below the close packing concentration [25] using the sequence of physical processes (SPP) analysis. Being developed by Rogers [9,29,30,31,32,33], this technique allows for connecting the time-resolved response of the sample to increasing shear deformations with the microstructural rearrangements happening during each cycle of deformation [32], thus revealing any changes that the additive might induce on the aggregation of Carbopol microgels. Finally, the results obtained through the rheological analysis were compared with the direct observation of the macroscopic structure of the Carbopol dispersions obtained via fluorescence microscopy.

## 2. Materials and Methods

### 2.1. Chemicals

Carbopol 974P NF (C974P manufactured by Lubrizol Ltd., Chertsey Surrey, UK) was donated by GlaxoSmithKline Consumers Healthcare (Middlesex, UK). This compound is classified as a highly crosslinked polyacrylic acid polymer [23] and, as such, is expected to maintain a well-defined particle identity even when swollen. In the unswollen state, the powder has apparent density $\rho_{p}=1.24$ g/cm${}^{3}$ and mean hydrodynamic radius $R_{i}=137$ nm, as experimentally determined in our previous study [25]. Poly(ethylene glycol) (PEG400 $M_{W}=400$ Da), glycerol (purity 99%), and D-Mannitol (ACS reagent, for microbiology, ≥99%) were purchased from Sigma–Aldrich (Dorset, UK). For fluorescence microscopy imaging, C974P chemically labelled with Rhodamine 123 (R123 mitochondrial specific fluorescent dye, Sigma Aldrich) was provided by the group of Prof. Paul Bartlett (School of Chemistry, University of Bristol, Bristol, UK).

### 2.2. Samples Preparation

Non-aqueous C974P dispersions at different polymer mass fractions were prepared following the same procedure that was reported in previous works [24,25]. In brief, independently of the specific sample composition, the polymer powder was first dispersed in PEG400 using a high-shear mixer (Silverson, L5 series) spinning at 8000 rpm. During this step, the temperature was constantly monitored and kept below 20 °C to inhibit particle swelling. Subsequently, the concentrated dispersions were diluted with the proper amount of PEG400 and glycerol to reach the final concentration required. Because C974P molecules swell more slowly in PEG400 than in glycerol, this procedure ensures a homogeneous dispersion of the particles in the liquid phase before any significant swelling has occurred. For the samples containing D-Mannitol, part of the glycerol was substituted with a stock solution of D-Mannitol at 4.2% wt in glycerol, which is below the solubility threshold of 5.5% wt [34]. To facilitate the dissolution of mannitol, glycerol was heated up to 40 °C with a stirred hot plate before the powder was gradually poured in. After dilution, the solutions were gently mixed in small batches of 30 g until being fully homogenized (∼10 s); then, the vials were sealed and left overnight in an ultrasonic bath (SciQuip Ultrasonic bath, 150 W) at 60 °C. The final solvent composition for all cases was of 70% wt/30% wt glycerol/PEG400 (solvent density $\rho_{s}=1.21$ g/cm${}^{3}$; solvent viscosity $\eta_{s}=0.707$ Pa s). For the same range of Carbopol concentrations, we prepared three sets of samples containing: (i) no mannitol (as a benchmark), (ii) 1.46% wt, and (iii) 2.87% wt of mannitol. When perfectly dissolved in solution, the amount of mannitol used did not significantly alter the densities and viscosities of the solvents. For brevity, from here on we refer to the three solutions as M0 (no mannitol), M1 (1.46% wt of mannitol), and M2 (2.87% wt of mannitol).

**Figure 1:**
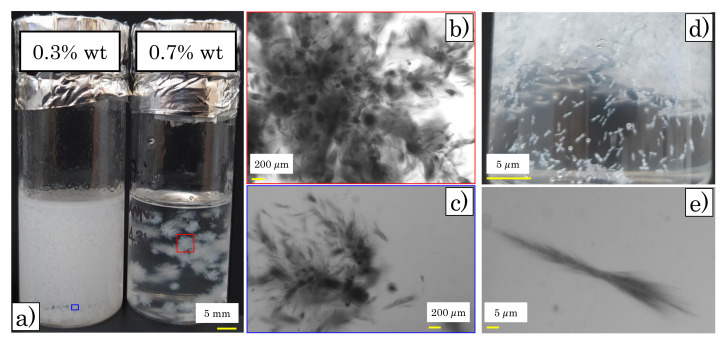
(**a**) Images of samples M2 at Carbopol concentrations of 0.3% wt (left) and 0.7% wt (right) taken after two weeks from sample initial preparation. (**b**–**c**) Brightfield images (upright optical microscope, Zeiss Axio Scope.A1) of a crystal isolated from the samples M2 at 0.7% wt (**b**) and at 0.3% wt (**c**) of Carbopol. (**d**) Detail of sample M1 at Carbopol concentration of 0.9% wt. (**e**) Brightfield image of a single crystal isolated from sample M1 reported in panel (**d**).

Upon completing the procedure described above, all of the samples appeared to be uniform and transparent for several days. Nonetheless, after some time all of the samples containing mannitol started to show small crystals suspended in solution (Figure 1). The kinetics of the crystallization phenomenon appeared to be sensitive to the amount of Carbopol and mannitol in solution. For the highest concentration of mannitol (samples M2), dilute samples of Carbopol ($w_{c}$ = 0–0.3% wt of Carbopol) showed, after two weeks, a higher number of small crystals, whilst, at intermediate concentrations ($w_{c}$ = 0.4–0.9% wt of Carbopol), the crystals were fewer and bigger. In both cases, the smallest elements of the crystals presented a “needle” shape, but the entire structure was partially different, with the bigger crystals presenting a star-like conformation, with denser cores and lateral arms, as can be seen in Figure 1a,b. For all samples with lower mannitol concentration (samples M1), small rod-like crystals formed in solution independently of the Carbopol concentration (Figure 1d,e). In all cases, for Carbopol concentrations close to and higher than the jamming concentration ($w_{c}>1$% wt [25]), the crystals were less distinguishable and the samples were opaque. All of the samples could be regenerated and returned to their initial transparent aspect by heating the dispersions at 60 °C for approximately 15 min. Once regenerated, the samples were stable for several days and, in some cases, for weeks. On the other hand, controlling the kinetics of the crystallization phenomenon was problematic. For the same sample, the formation of crystals after the regeneration process can vary significantly in time.

Although crystallization of D-mannitol in water solutions has been reported by several authors [35,36,37], these observations highlight new interesting crystallization phenomena in the absence of water, which appear to be influenced by the steric hindrance of Carbopol particles dispersed in solutions. However, since this work primarily aimed to investigate the effect of the additive (i.e., mannitol) on the rheological properties of Carbopol dispersions, we focused on the rheological properties of the samples with no visible crystals in solution and with the crystals, postponing the study of the crystallization phenomenon to possible future work.

### 2.3. Rheological Measurements

An Anton Paar MCR302 was used to perform all of the rheological measurements. The instrument was equipped with a Peltier plate to control the operating temperature and a parallel plate (OD: 40 mm) with sandblasted surfaces to avoid slip effects (surface roughness of 2 μm). The consistency of the gap was maintained throughout all of the tests (gap height of 900 μm). All of the measurements were performed at an ambient temperature of 25 °C. Given the difficulty of controlling the kinetics of crystallization, all of the samples were first tested after two days from the initial preparation, and then tested after three weeks to give time to the crystals to form, and then tested again after regeneration. The results obtained after two days agree with those obtained after regeneration, thus confirming the efficacy of the regeneration process. Before each test, the samples were pre-sheared at a constant shear rate of 50 1/s for 60 s and then left to rest for 30 min. to remove any mechanical history that is caused by the loading procedure. Because the addition of mannitol could potentially influence the osmotic equilibrium in solution, thus affecting the swollen dimension of Carbopol particles at equilibrium, we first conducted steady-shear tests on dilute and semi-dilute Carbopol dispersions without (M0) and with (M1 and M2) mannitol. For the latter, we only considered fresh or regenerated solutions to ensure that crystals were absent. The tests were carried out sweeping the shear rate $\dot{\gamma }$ from 0.001 to 500 1/s and recording the shear stress σ once the torque signal had become steady. The Weissenberg–Rabinowitsch correction [38] for parallel-plate geometry was applied to all data acquired.

In order to investigate the effect of the additive on the microstructure of the material, we conducted small amplitude oscillatory tests (SAOS) by sampling the viscoelastic response of the samples in the linear regime of deformations, determining the storage (G${}^{\prime }$) and loss (G${}^{\prime \prime }$) moduli. The tests were performed applying a sinusoidal shear strain γ with a fixed amplitude of 0.006 and angular frequency spanning the range ω = 100–0.01 rad/s and repeated for all solvent compositions with and without crystals. All of the samples with concentrations close to the threshold at which the liquid-to-solid transition occurs were then investigated via large amplitude oscillatory sweeps (LAOS).

The viscoelastic response to large sinusoidal deformations of a microgel system below and above the jamming point can provide great insight into the physical mechanism that regulates the yielding of the material and, therefore, into its microstructure. The tests were performed applying a sinusoidal deformation, being characterised by a constant angular frequency of 1 rad/s and varying the amplitude in the range of $10^{-4}$–$10^{2}$. For each amplitude, the sinusoidal oscillations were maintained until the periodic signal became stable; at this point, the time-resolved data were collected for few periods of oscillation. The strain, shear rate, and stress waveforms that were acquired in this fashion were then analysed following the sequence of physical processes (SPP) analysis. This technique, which was developed by Rogers [9,29,30,31,32,33], provides a unique way to physically interpret raw data that were acquired from LAOS tests, thus unveiling the physical processes underlying the yielding phenomenon. All of the data were processed using the MATLAB-based SPPplus v2 software [9], which was kindly provided by Prof. Rogers. The program first reconstructs the data via Fourier-domain filtering, using all of the detectable odd harmonics to reduce any instrument noise, and then applies the SPP framework, which is briefly described in the following section.

### 2.4. Sequence of Physical Processes (Spp) Analysis

The SPP analysis employs the description of the stress response as a function of both the strain and shear rate to extract information on the elastic and viscous contributions to the response of the material when an external deformation is applied. Contrary to other established frameworks, such as Fourier transform rheology [38,39], Chebyshev polynomial expansion [40], and other stress and waveform decomposition approaches [41,42], which are mathematically robust, but lack a clear and complete physical interpretation, the SPP framework provides an unambiguous physical interpretation of the stress signals for any type of material without making any assumptions on the symmetries of the response. A full description of the mathematical derivation of the approach can be found in the original work that was developed by Rogers [30]; here, we summarise the basic concepts, which are necessary for interpreting our experimental results.

**Figure 2:**
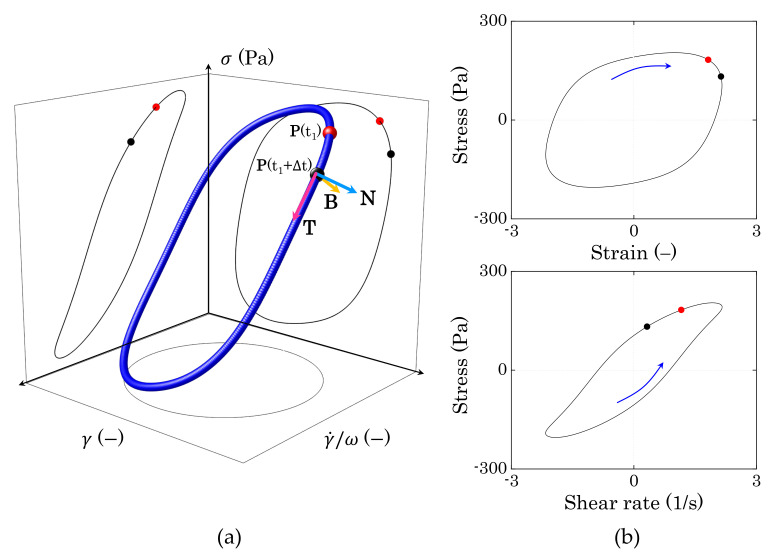
(**a**) Example of the nonlinear stress response to a periodic sinusoidal deformation reported in the three-dimensional space [γ, $\dot{\gamma }/\omega$, σ]. Each position vector P(t) on the curve can be identified through the Frenet–Serret components, as reported in figure. (**b**) Projections of the three-dimensional trajectory on the [γ, σ] and [$\dot{\gamma }/\omega$, σ] planes (Lissajous–Bowditch plots). The blue arrows indicate the orientation of the trajectories.

For a complex material, which behaves neither as a linear elastic solid nor as a viscous liquid, the stress response σ(t) to a periodic deformation is a function of both the deformation applied γ(t) and the shear rate induced by the external movement of the upper plate of the rheometer $\dot{\gamma }\left(t\right)$. Hence, for a single period of deformation, the stress can be visualized in the three-dimensional (3D) space [γ(t), $\dot{\gamma }\left(t\right)/\omega$, σ(t)], where $\dot{\gamma }\left(t\right)/\omega$ is the instantaneous shear rate normalised against the characteristic angular frequency of the oscillation imposed, as shown in Figure 2a. The projections of the three-dimensional (3D) curve on the [γ(t), σ(t)] and [$\dot{\gamma }\left(t\right)/\omega$] planes give the classic Lissajous–Bowditch curves [40,43] used to visualize the intracycle elastic and viscous contributions of the sample response to the applied deformation, respectively (Figure 2b).

The dynamic evolution of the 3D trajectory of the stress curve varies with the specific physical processes that are responsible for the material response. Hence, the difference in local orientation of the stress curve between two position vectors P(t) and P(t+Δt) can provide information on the evolution of the internal microstructure of the material during the deformation cycle. In the limit of infinitesimal time steps (Δt→dt), the stress variation is given by:

(1) $$d\sigma =\frac{\partial \sigma }{\partial \gamma }d\gamma +\frac{\partial \sigma }{\partial \left(\dot{\gamma }/\omega \right)}d\left(\dot{\gamma }/\omega \right).$$

The derivatives on the right-hand side of Equation (1), $\partial_{\gamma }\sigma$, and $\partial_{\dot{\gamma }/\omega }\sigma$ represent the terms in-phase and out-of-phase with the deformation γ(t), respectively, and are, therefore, defined as the instantaneous storage and loss moduli, $G_{t}^{\prime }\left(t\right)$ and $G_{t}^{\prime \prime }\left(t\right)$, in analogy with the definitions of G${}^{\prime }$ and G${}^{\prime \prime }$ in the linear viscoelasticity theory, with which they coincide in the limit of infinitesimal deformations. The instantaneous moduli are defined as partial, not total, derivatives of the stress against the strain and shear rate and, thus, permit to separate exactly the elastic from the viscous contributions. This approach differs from that of Lissajous and Bowditch, which considers the total derivatives of the stress ($d\sigma /d\gamma =\partial_{\gamma }\sigma +\partial_{\dot{\gamma }}\sigma \left(d\dot{\gamma }/d\gamma \right)$ and $d\sigma /d\dot{\gamma }=\partial_{\dot{\gamma }}\sigma +\partial_{\gamma }\sigma \left(d\gamma /d\dot{\gamma }\right)$) and, therefore, cannot isolate the dependence of σ on γ and $\dot{\gamma }$.

Within the SPP framework, the instantaneous moduli $G_{t}^{\prime }\left(t\right)$ and $G_{t}^{\prime \prime }\left(t\right)$ are extracted by using the Frenet–Serret frame to describe the three-dimensional trajectory of the stress. In brief, each point of the curve $\sigma [\gamma \left(t\right),\dot{\gamma }\left(t\right)]$ is identified by a position vector P(t), which can be decomposed using the Frenet–Serret frame. This is the orthonormal vector basis that is formed by (i) the tangent vector T(t) (the unit vector tangent to the curve in the direction of the motion), (ii) the normal vector N(t) (the derivative of T with respect to the arc-length parameter of the curve), and (iii) the binormal vector B(t) (the cross product between T and N). Following the original mathematical derivation [9,30], the two instantaneous moduli are defined as:

(2) $$G_{t}^{\prime }\left(t\right)=-\frac{B_{\gamma }\left(t\right)}{B_{\sigma }\left(t\right)}.$$

(3) $$G_{t}^{\prime \prime }\left(t\right)=-\frac{B_{\dot{\gamma }/\omega }\left(t\right)}{B_{\sigma }\left(t\right)}.$$

where $B_{\gamma }$, $B_{\dot{\gamma }/\omega }$, and $B_{\sigma }$ are the projections of the binormal vector along the coordinate directions γ, $\dot{\gamma }/\omega$ and σ, respectively. As the position vector moves along the stress trajectory, the orientation of B(t) changes accordingly, thus changing its projections.

**Figure 3:**
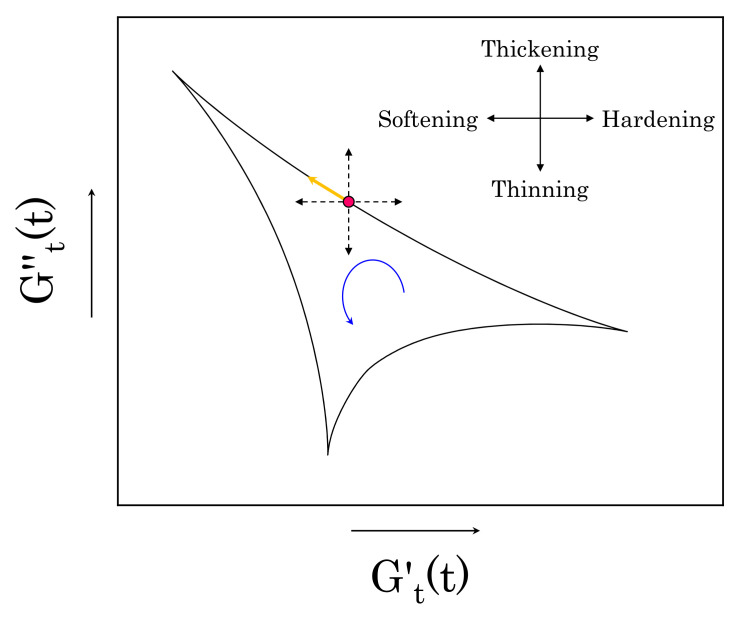
Schematic of the rheological transitions that can be observed using the Cole–Cole plot. As an example, we highlight the instantaneous trajectory of a point on the curve (yellow arrow). In that part of the cycle, the instantaneous storage modulus is decreasing, while the instantaneous loss modulus is increasing; this indicates a simultaneous softening and thickening of the material.

Accordingly, all of the rheological transitions that take place during a deformation cycle are described by the change in the two instantaneous moduli, which, for each cycle, can be more conveniently reported in the Cole–Cole plot, whose abscissa and ordinate are given by $G_{t}^{\prime }\left(t\right)$ and $G_{t}^{\prime \prime }\left(t\right)$, respectively [9,30,31,44]. This representation allows for following the evolution of the two parameters during each strain cycle, offering a simple rheological interpretation. Starting from any point on the curve, motions along the horizontal coordinate indicate elastic changes, stiffening for an increase in $G_{t}^{\prime }\left(t\right)$ and softening otherwise; similarly, motions along the vertical coordinate indicate a viscous rheological transition, thickening for an increase of $G_{t}^{\prime \prime }\left(t\right)$ and thinning otherwise [31,44]. Figure 3 shows a schematic of the transitions in the Cole–Cole space. Regarding the Lissajous–Bowditch plots, the trajectories in a Cole–Cole plot always follow the same orientation (anticlockwise). Note that, in the limit of infinitesimal strain amplitudes, the plot would collapse into a single point with coordinates equal to G${}^{\prime }$ and G${}^{\prime \prime }$, indicating the absence of any intracycle microstructural rearrangements for infinitesimal deformations.

## 3. Results

### 3.1. Swollen Particle Size

**Figure 4:**
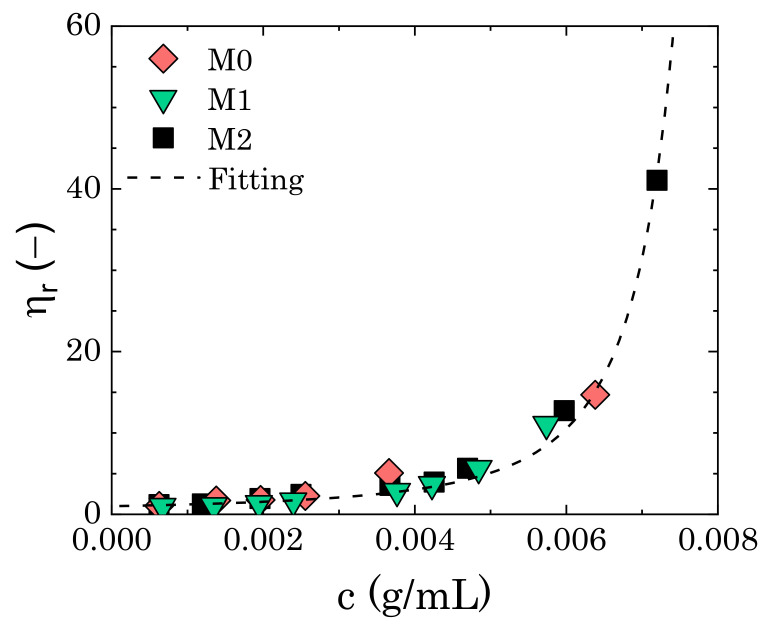
Relative zero-shear viscosity as a function of Carbopol concentration for mannitol contents of 0% (M0), 1.46% wt (M1), and 2.87% wt (M2). The dashed line is the fitting with Mooney’s equation.

We first assessed the effect of mannitol on the final swollen dimensions of Carbopol particles by analysing the trend of the relative zero-shear viscosity (i.e., $\eta_{r}=\eta_{0}/\eta_{s}$, where $\eta_{0}$ is the viscosity plateau of the flow curves at small shear rates) with Carbopol concentration (*c*) in the three different solvents. Figure 4 shows the results. Independently of the content of mannitol, the data points overlap well, thus indicating that, when perfectly dissolved in solution, i.e., in the absence of crystals, mannitol does not influence the final swollen dimension of the Carbopol particles. Assuming a linear relation between the Carbopol mass concentration and volume fraction ϕ (i.e., c=kϕ, where *k* is a proportionality constant), the data shown in Figure 4 can be fitted using the Mooney equation for the viscosity of concentrated particle suspensions [45]:

(4) $$\eta_{r}=\exp\left(\frac{2.5\phi }{1-\lambda \phi }\right)=\exp\left(\frac{2.5kc}{1-\lambda kc}\right).$$

In Equation (4), λ is a fitting parameter that depends on the maximum packing volume fraction ($\phi_{MP}$) that the specific particle system can achieve (i.e., $\lambda \approx 1/\phi_{MP}$). In a previous work [25], we found a value of λ equal to 1.3 for the same dispersions. By substituting this value in Equation (4), the fitting gives a proportionality constant k=70.54 g/mL, which, in turn, gives a final swollen particle radius $R_{SW}=R_{i}{\left(\rho_{p}k\right)}^{1/3}\approx 608$ nm, a value that is consistent with dimensions previously found in dispersions of the same Carbopol molecule in pure glycerol or a mixture of glycerol and PEG400 with 50% wt of glycerol [25].

These results suggest that, even in the presence of mannitol, the effect of glycerol on the final dimension of the Carbopol particles at equilibrium still dominates over the other components in the solution, and the addition of another polyol does not create significant osmotic imbalance, owing to the similar chemical nature of mannitol and glycerol molecules. Hence, we can safely assume that any difference that may arise in the next sections in the rheological behaviour of the dispersions in the three solvents stems not from a difference in the effective volume fraction of the Carbopol particles, but rather from a difference in the interactions and overall microstructure of the sample.

### 3.2. Linear Viscoelastic Properties

**Figure 5:**
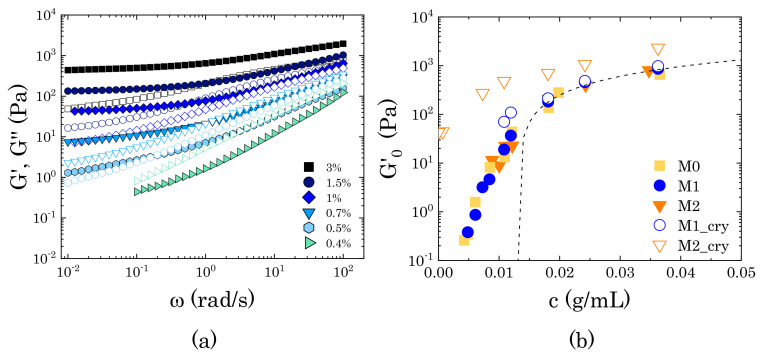
(**a**) Frequency dependence of the storage (closed symbols) and loss (open symbols) moduli at increasing Carbopol concentration. The data refer to samples M0, but the same behaviour was observed for regenerated M1 and M2 solutions. (**b**) Storage modulus, sampled at 1 rad/s, as a function of Carbopol concentration for samples M0, M1, and M2. For the samples containing mannitol, closed symbols indicate regenerated samples, while open symbols indicate samples containing crystals. The black dashed line represents the fitting with the linear relation $G_{0}^{\prime }=K_{p}\left(c-c_{c}\right)$ reported in [25] for the same Carbopol dispersions in pure glycerol. A table reporting the effective mass percentages and corresponding mass concentration for each sample (i.e., M0, M1, and M2) is reported in the  Supplementary Materials (Table S1).

In the absence of crystals, the viscoelastic response in the limit of linear deformations remains unchanged for all of the solutions. As the Carbopol concentration is increased, the solutions transition from a viscous liquid to a jammed glass (Figure 5). At low Carbopol mass fraction (0.4% wt), the loss modulus is slightly larger than the storage modulus in the entire range of frequencies sampled, even though the similar slopes of G${}^{\prime }$ and G${}^{\prime \prime }$ (slope ∼0.9 Pa s) indicate the presence of local relaxation phenomena induced by local rearrangements of the particles in solution [46]. As the concentration of Carbopol increases, particle confinement becomes more significant and the macroscopic viscoelastic response changes accordingly. G${}^{\prime }$ increases and its slope gradually decreases at low frequencies. At intermediate Carbopol concentrations, this causes a crossover with G${}^{\prime \prime }$, where G${}^{\prime }$ slightly overtakes G${}^{\prime \prime }$ in the low range of frequencies (0.5%). This region gradually expands at higher frequencies, until reaching an almost constant value of G${}^{\prime }$ for the entire range sampled (3% wt).

The loss modulus also gradually increases with polymer concentration, although showing a different evolution when compared to G${}^{\prime }$. The storage modulus depends on the elasticity of the whole network of particles, which gradually increases with particle density until reaching a plateau value that is related to the elasticity of the single microgel particle [47,48,49]. For the loss modulus, the response is more complex and it arises from: (i) the viscous dissipation induced in the liquid solvent by the external deformation applied, a contribution that increases monotonically with the frequency independently of the particle concentration and (ii) the viscous dissipation that is induced by the motion of the particles. This second contribution is associated with particle mobility; hence, it depends on the relaxation phenomena that are associated with particle motion and changes with an increase in Carbopol concentration. This entails that, as confinement gradually restricts particle movement, the characteristic relaxation time of the particle motion increases and the contribution to the viscous dissipation rises at low frequencies, thus showing a decrease in the slope of G${}^{\prime \prime }$ in the low-frequency range, but maintaining a similar slope at higher frequencies. As the Carbopol concentration increases, the polymer particles are gradually forced to contact and, thanks to their softness, they can deform against each other, thus forming a densely packed system of soft particles. In these conditions, the viscoelastic response is controlled by the balance between the elasticity of the packed particles and the viscous dissipation induced by particles sliding against each other [50,51,52]. Consequently, the loss modulus continues to rise, maintaining values in the order of $10^{2}$, but the gap between G${}^{\prime }$ and G${}^{\prime \prime }$ increases, and the storage modulus dominates over the loss modulus in the entire range of frequencies (3% wt in Figure 5a).

The transitions that are described above can also be visualized by plotting the evolution of the storage modulus with Carbopol concentration at a constant frequency of 1 rad/s ($G_{0}^{\prime }$). The results are reported in Figure 5b. In the absence of crystals, the data points fall on the same curve, thus indicating that, when perfectly dissolved in solution, mannitol does not influence the phase transition that is experienced by the dispersions. Starting from the lowest Carbopol concentrations, $G_{0}^{\prime }$ first increases steeply with a slope controlled by the soft interparticle interactions [50,53,54,55], and then smoothly transitions to a linear asymptotic trend, as commonly observed for densely packed microgel systems [48,49,50,54,56]. Although a unified physical picture is not available, this trend is typically associated with the ability of soft particles to adapt their shape as they are pushed against each other at increasing polymer concentration. In these conditions, the elastic behaviour is controlled by the physical properties of the microgel core, which gradually densifies as the particle concentration increases, resulting in a linear increase of the storage modulus plateau [50,57]. This trend is typically described as a linear function of the particle volume fraction,

(5) $$G_{0}^{\prime }=G_{p}\left(\phi -\phi_{c}\right).$$

where $G_{p}$ is the particle elastic modulus and $\phi_{c}$ is the characteristic volume fraction corresponding to close packing conditions, i.e., the volume fraction at which particle deformations become significant. Assuming the same linear approximation considered in Section 3.1 (i.e., ϕ≈kc), Equation (5) can be expressed in terms of Carbopol concentration as $G_{0}^{\prime }=k_{p}\left(c-c_{c}\right)$, where $k_{p}\approx kG_{p}$ is a proportionality constant linearly dependent on the particle elastic modulus and $c_{c}$ is the Carbopol concentration corresponding to $\phi_{c}$. Since the data points in the densely packed regime are limited in this study, we report in Figure 5b the linear fitting obtained from our previous study with the same Carbopol dispersions in pure glycerol [25]. The fitting was obtained using Carbopol mass fractions up to 8% wt, yielding $k_{p}=38$ m${}^{2}/$s${}^{2}$ and $c_{c}=0.0158$ g/mL. As can be seen, in the absence of crystals, the storage modulus of the dispersions at concentrations above close packing falls onto the linear fitting, thus indicating that, for those solutions, the elastic behaviour is controlled by the elasticity of the specific Carbopol molecules [58].

**Figure 6:**
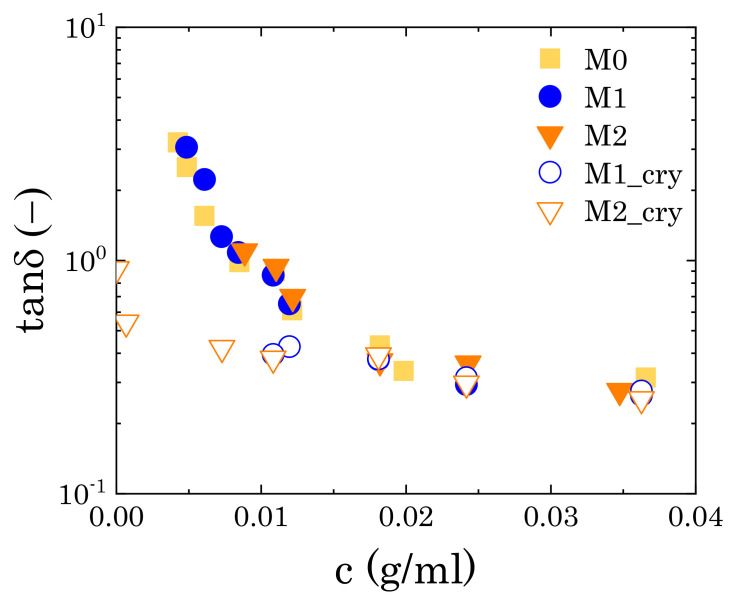
Loss tangent, sampled at 1 rad/s, as a function of Carbopol concentration for samples M0, M1, and M2. For the samples containing mannitol, closed symbols indicate regenerated samples, while open symbols indicate samples containing crystals.

In the presence of crystals, the elastic character of the dispersions increases. For M1 solutions, the increase of $G_{0}^{\prime }$ at the highest concentrations is almost negligible, but it reveals itself at intermediate concentrations, as shown by the deviation from the steep trend followed by the closed symbols shown in Figure 5b. For M2 solutions, the storage modulus increases further and maintains a finite value, even in the absence of Carbopol (i.e., c=0). At a high concentration, the increase in elasticity is not followed by a decrease of the viscous component, as would happen in the case of the formation of a macroscopic interconnected structure (i.e., macrogel), as can be observed by the trends of the loss tangent (tanδ≡ G${}^{\prime \prime }$/G${}^{\prime }$) at the same angular frequency (Figure 6). This indicates that in the presence of crystals the solutions preserve the features typical of particulate suspensions and the viscoelastic properties are predominantly controlled by the local rearrangements of the Carbopol particles at close contact. As Carbopol concentration reduces, the impact of the crystals becomes more relevant, until the system reaches a condition where the viscoelastic response is only controlled by the local rearrangements of the crystals in solutions, as shown by the high values of G${}^{\prime }$ for c→0. Therefore, the addition of mannitol seems to alter the rheological properties of the dispersions only when a second dispersed phase forms in solution (i.e., crystals), thus interfering with the local dynamics of Carbopol particles.

### 3.3. Nonlinear Viscoelastic Properties

#### 3.3.1. Average Viscoelastic Properties

We further investigated the response of the dispersions to large amplitude oscillatory deformations to obtain a clearer picture of the nature of the interactions between Carbopol particles and the crystalline phase dispersed in solution. The behaviour of two samples with Carbopol concentrations above (2% wt) and below (0.9% wt) the close packing concentration $c_{c}$ is compared for the three solvent compositions. Note that from this point onward all data reported for samples containing mannitol refer to samples with a visible crystalline phase; when regenerated, samples M1 and M2 present the same rheological properties of sample M0 and, therefore, are not reported in the following analysis.

**Figure 7:**
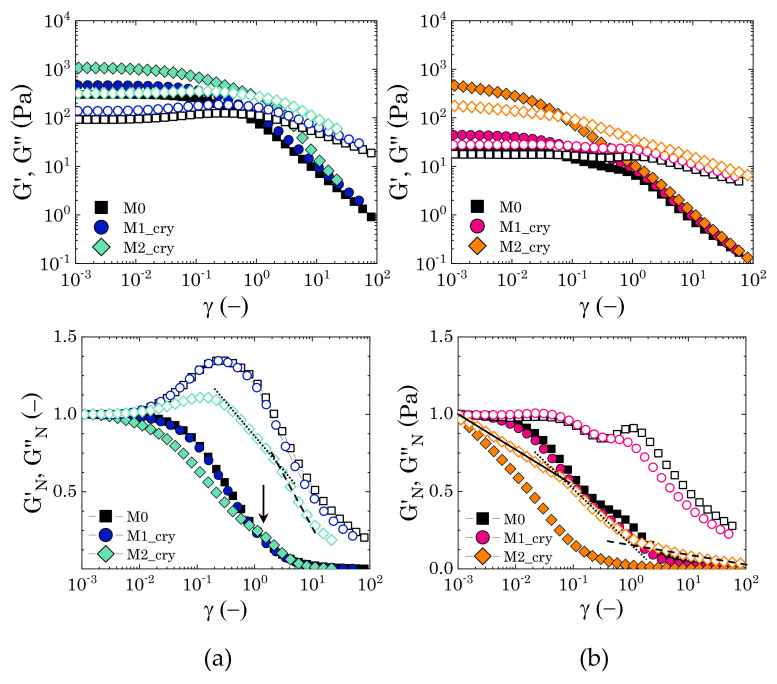
(**a**) Strain amplitude dependence of the storage (closed) and loss (open) moduli (upper panel) and of the corresponding normalized quantities (bottom panel) at ω=1 rad/s for a Carbopol mass fraction of 2% wt in the absence of mannitol (M0) and for the two mannitol concentrations considered, in the presence of crystals. The black arrow points to the local maximum of $G_{N}^{\prime }$. (**b**) The strain amplitude dependence of the storage (closed) and loss (open) moduli (upper panel) and of the corresponding normalized quantities (bottom panel) at ω=1 rad/s for a Carbopol mass fraction of 0.9% wt in the absence of mannitol (M0) and for the two mannitol concentrations considered, in the presence of crystals. The black solid, dotted, and dashed lines are guides for the eye to indicate the two different decays, followed by $G_{N}^{\prime \prime }$.

The presence of crystals influences the viscoelastic response of the dispersions both quantitatively and qualitatively, as can be observed in Figure 7. At higher Carbopol concentrations (left panels), the typical behaviour of a packed suspension of soft particles is observed for all solutions: G${}^{\prime }$ dominates over G${}^{\prime \prime }$ in the linear regime of deformations, but it gradually decreases at higher strain amplitudes, until showing a crossover with G${}^{\prime \prime }$. In this region of strain amplitudes, the elastic restoring force is overcome by the external stress that is induced by the deformation applied and the material yields [12,28]. The crossover is typically accompanied by a peak of G${}^{\prime \prime }$, attributed to an increase of the viscous dissipative energy associated with particle rearrangement [9,59], and is followed by a power law decrease of both moduli. At first sight, the presence of mannitol crystals seems to only influence the magnitude of the two moduli, even though, for sample M2, the two moduli decay with a different power law as compared to the other two samples. Hence, to better reveal the differences, we replotted the same data normalized by their plateau values, i.e., $G_{N}^{\prime }=$G${}^{\prime }$/G${}_{\gamma \to 0}^{\prime }$, $G_{N}^{\prime \prime }=$G${}^{\prime \prime }$/G${}_{\gamma \to 0}^{\prime \prime }$ (bottom panels in Figure 7). The single peak of the loss modulus is revealed for samples M0 and M1, which show a good superposition of both normalized variables, while the data for sample M2 deviate from the common trend. In the latter case, G${}^{\prime \prime }$ shows a less pronounced peak at the same strain amplitude as the other two samples (γ∼0.2), followed at first by a less pronounced decay, up to γ∼2, and after by a decay similar to that of the other two solutions. Note that just before the end of the first decay (i.e., γ close to 2), $G_{N}^{\prime }$ presents a small local maximum (as indicated in Figure 7a by the black arrow), which could be related to an accumulation of elastic energy in the internal microstructure, which is then viscously dissipated when the sample is sheared at higher strain amplitudes [19,60].

For Carbopol concentrations below $c_{c}$, particle confinement is still significant, but particles are not deformed against each other and the solvent gradually occupies larger interstitial spaces between them. In this particle concentration regime, the flow behaviour is not only controlled by the balance between the elastic restoring force and the viscous sliding between the squeezed microgels, but the nature of the interparticle interactions and the presence of heterogeneities in the microstructure begins to play a crucial role in the flow behaviour of the dispersions. For a Carbopol concentration of 0.9% wt, in the absence of mannitol, the initial gap between G${}^{\prime }$ and G${}^{\prime \prime }$ reduces, and the peak of G${}^{\prime \prime }$ at the crossover is substituted by a local depression, followed by a local peak at higher strain amplitudes (γ∼2) and a mild shear-thinning decrease, as can be observed from Figure 7b. The corresponding storage modulus follows the typical linear trend at low strain amplitudes and a shear-thinning decay at higher strains; however showing a local peak just before the local maximum of G${}^{\prime \prime }$. A similar trend is observed for sample M1, even though the normalized moduli reveal an initial small peak around γ∼0.03, where the two moduli crossover, followed by a first decay plateauing at γ∼1 and a second one at the highest strain amplitudes, which preserves the same trend that was observed in sample M0. On the other hand, in the presence of a well-structured crystalline phase, as in the case of sample M2 (Figure 1a), the response to increasing strain amplitudes is altered. The linear viscoelastic region reduces significantly, with both moduli showing a decreasing profile since the lowest amplitude sampled. Furthermore, both normalized moduli show a strain-thinning trend [43], with the loss modulus presenting a three-stage decay with no significant overshoot.

All of the trends observed at this concentration regime highlight a complex sequence of processes leading to the flow of the dispersions, which is directly connected to the disruption of their internal microstructure. In the first two cases (M0 and M1), the presence of two mild overshoots of the loss moduli is typically associated with the viscous dissipation that is caused by the rupture of particle aggregates at multiple length scales [59,60]. The first overshoot is usually associated with the rupture of direct particle-particle contacts, which allows greater mobility, thus inducing a temporary decrease of the viscous contribution; the second is related to the rupture of bigger structures, which ensures the complete flow of the dispersions. On the other hand, for sample M2, the stronger elastic character, as revealed by higher values of the storage modulus and the three-stage decay of the loss modulus, indicates that the presence of crystals disrupts the “cage” structure typical of Carbopol dispersions close to the jamming point [25]. This probably generates a more heterogeneous, but interconnected, structure that strengthens the structural stability of the dispersions at vanishing strains, but undergoes a sequential de-structuring at increasing strain amplitudes.

The storage and loss moduli only unambiguously represent the elastic and viscous contributions in the linear regime of deformations. Thus, to obtain a clearer physical picture of the flow behaviour that is reported in Figure 7, we further investigated the two extreme cases, i.e., samples M0 and M2, by studying the time-dependent response of the stress during a full cycle of the sinusoidal deformation. The results were then analysed while using the SPP framework.

#### 3.3.2. Evolution of the Instantaneous Moduli for $c>c_{c}$

**Figure 8:**
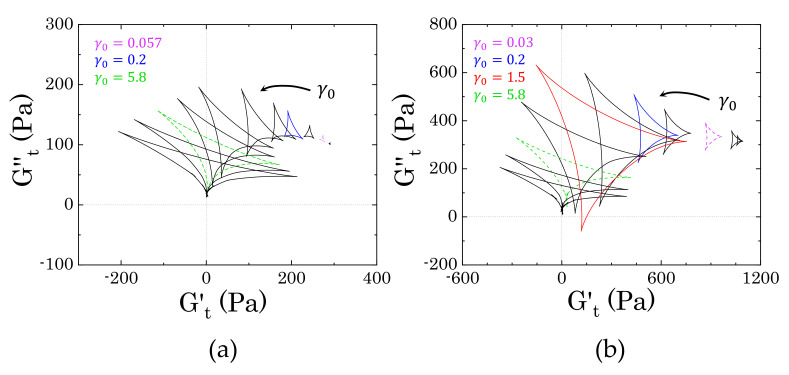
Evolution of the Cole–Cole plots at increasing strain amplitudes for two samples at 2% wt of Carbopol (**a**) in the absence of mannitol (sample M0) and (**b**) with mannitol in the presence of crystals (sample M2). All of the deltoids corresponding to the most relevant transitions observed in Figure 7a are marked with a specific colour. Dashed purple line: first point deviating from the linear viscoelastic plateau; blue solid line: peak of G${}^{\prime \prime }$; red solid line: end of the first decay of G${}^{\prime \prime }$ for sample M2; green dashed line: beginning of final strain-thinning behaviour.

Starting from the more concentrated sample (2% wt Carbopol), Figure 8 shows the evolution of the trajectories of the instantaneous moduli (Cole–Cole plots) at increasing strain amplitudes $\gamma_{0}$. In both cases, the Cole–Cole plots present the typical deltoid shape, deriving from a dominance of the third harmonic of the Fourier series used to reconstruct the stress signal [31,32], but the orientation and the area of the cycles progressively change as $\gamma_{0}$ increases. These two features, i.e., orientation and area of the cycle, describe the nature of the sequence of physical processes that the material experiences during each cycle, therefore providing insights into the microstructural rearrangements induced by the external deformation applied. Specifically, the orientation of the trajectories indicates the nature of the transition, as already mentioned in Section 2.4, while the area relates to the range of intracycle rheological transitions occurring in the material: the larger the area, the wider the range of structural rearrangements of the material [31].

The results obtained from the two different samples show a clear difference in the progression of the cycles, thus indicating that the two dispersions experience different intracycle rheological transitions and, therefore, present different internal microstructures. The first clear discrepancy is the dimensions of the deltoids, which are significantly larger in sample M2 (Figure 8b) and they follow a non-monotonic increase with the strain amplitude, as opposed to sample M0, which shows a gradual increase of the areas of the trajectories. These characteristics indicate that, in the absence of mannitol, the structural rearrangements that the packed dispersions experience are more limited and gradually progress with an increase of the strain amplitude, thus suggesting that the main structural unit does not change during the yielding process. On the other hand, in the presence of crystals, the material experiences different structural rearrangements, which might be related to a more heterogeneous structure that breaks down as the yielding proceeds. To clarify the nature of the transitions, we compare the single trajectories that are representative of different regions of strain amplitudes. The regions of interest can be chosen accordingly to the main features of the profiles of G${}^{\prime }$ and G${}^{\prime \prime }$ reported in Figure 7. Specifically, we consider (i) the initial deviation from the linear viscoelastic region, marked in purple in Figure 8, (ii) the first peak of the loss modulus, marked in blue, and (iii) the strain-thinning region following the peak.

**Figure 9:**
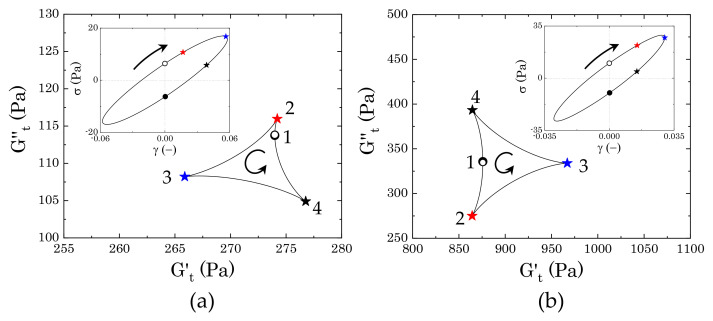
(**a**) Cole–Cole plot obtained at $\gamma_{0}=0.057$ (beginning of the deviation from the linear viscoelastic region) for sample M0 at 2% wt of Carbopol. (**b**) Cole–Cole plot obtained at $\gamma_{0}=0.03$ (beginning of the deviation from the linear viscoelastic region) for sample M2 at 2% wt of Carbopol. The insets show the corresponding elastic Lissajous–Bowditch plot (i.e., stress vs. strain) for reference.

The difference between the two samples arises since the first deviation from the linear viscoelastic regime, as can be seen from the trajectories reported in Figure 9. In the absence of mannitol (Figure 9a), the dispersions experience a very mild intracycle restructuring, as reflected by the small range of values spanned, which are close to the values found for the storage and loss moduli (at $\gamma_{0}=0.057$, G${}^{\prime }$ =272.3 Pa and G${}^{\prime \prime }$ =109.7 Pa). As the strain gradually increases from zero to its maximum (from point 1 to 3), we initially observe an increase of the viscous contribution with a weak increase of the instantaneous elastic modulus (up to point 2), followed by a gradual decrease of both moduli, until $G_{t}^{\prime }$ reaches its minimum. From a microstructural viewpoint, this transition indicates that, to force the particles to slide against each other as the strain increases from the zero value, the initial contacts between the particles must be broken; this generates a surge of viscous dissipation (maximum of $G_{t}^{\prime \prime }$), accompanied by a mild bond stretching. Once this initial barrier is overcome, particles can slide more easily and both the viscous and elastic contribution decrease until the strain reaches its maximum value and the shear rate vanishes. At this point, the elastic contribution reaches its minimum. But as the strain gradually decreases, the instantaneous loss modulus decreases, reaching its minimum, whilst $G_{t}^{\prime }$ rises to its maximum (point 4). At point 4, the strain is approaching zero and the shear rate its maximum value, so the viscous contribution increases and the structure softens, showing a mild reduction of the elastic instantaneous modulus. The trajectories found suggest that the microstructure experiences a sequential process, with an initial structure recovery at the beginning of the strain reversal (from point 3 to point 4), where the initial packed cage reforms, followed by a subsequent increase of the viscous contribution as the shear rate increases towards its maximum (from point 4 to point 1).

In the presence of mannitol crystals, the intracycle rheological transitions change (Figure 9b). As in the previous case, the centre of the deltoid corresponds to the values of G${}^{\prime }$ and G${}^{\prime \prime }$ (at $\gamma_{0}=0.03$, G${}^{\prime }$ =898.9 Pa and G${}^{\prime \prime }$ =334.5 Pa), but, in this case, the material experiences wider ranges of structural transitions, as highlighted by the larger area covered by the trajectory. The qualitative response also appears to be different. As the strain evolves from zero to its maximum amplitude, the dispersions first experience softening and thinning of the structure, as indicated by the decrease of both $G_{t}^{\prime }$ and $G_{t}^{\prime \prime }$, which reach their minimum at point 2. This is followed by a rapid increase of the elastic instantaneous modulus, culminating in its maximum at the maximum strain amplitude, and a mild increase of the viscous instantaneous modulus. This trend suggests that the initial movement of the dispersed phases is accompanied by a softening and alignment of the internal structure (from point 1 to point 2), followed by a stretching, where the strain is mainly accumulated elastically (from point 2 to point 3). At the point of strain reversal, the stretching is released and the residual stress is dissipated viscously until the system reaches a maximum of the viscous instantaneous modulus (point 4). After this point, as the strain approaches zero, the viscous dissipation reduces and the structure partially strengthens before repeating the same cycle. Note that, aside from the differences in stress magnitude, the corresponding elastic Lissajous–Bowditch plots, reported as insets in Figure 9, are identical; this highlights how the use of the instantaneous moduli can offer insightful information, even in the low range of strain amplitudes.

**Figure 10:**
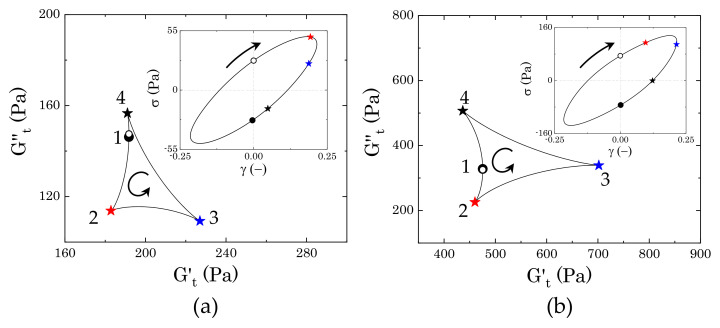
(**a**) Cole–Cole plot obtained at $\gamma_{0}=0.2$ (peak of the loss modulus) for sample M0 at 2% wt of Carbopol. (**b**) Cole–Cole plot obtained at $\gamma_{0}=0.2$ (peak of the loss modulus) for sample M2 at 2% wt of Carbopol. The insets show the corresponding elastic Lissajous–Bowditch plot (i.e., stress vs. strain) for reference.

As the strain amplitude $\gamma_{0}$ increases, the nonlinear character of the stress response to the sinusoidal deformation increases. Figure 10 reports the trajectories of the instantaneous moduli and corresponding elastic Lissajous–Bowditch plots at the strain corresponding to the peak of the loss modulus observed in Figure 7a.

In the absence of mannitol, the qualitative features of the instantaneous moduli are different from those that are shown in Figure 9a at lower amplitude strains. Figure 10a shows initial thinning and mild softening of the structure, as the strain increases from zero to its maximum. In this case, the initial rate of change of the strain applied (shear rate), which is maximum at point 1, is one order of magnitude higher than the previous case, thus causing a quicker disruption of the bonds between particles, which are suddenly forced to slide against each other. As the strain reverses, passing through its maximum point, the structure recovers, causing an increase of $G_{t}^{\prime }$ to its maximum and a further reduction of $G_{t}^{\prime \prime }$, which reaches its minimum (point 3). As the strain further reduces and the shear rate increases, the structure formed during the strain reversal softens, particles escape their positions by sliding against each other and the viscous contribution increases until reaching its maximum at point 4. As the strain approaches zero and the point of shear rate reversal, the viscous dissipation reduces and the elastic contribution maintains the same value until repeating the same cycle. On the other hand, in the presence of mannitol, the qualitative behaviour remains very similar to the previous case and the area of the trajectories enlarges, thus indicating that the same transitions described previously for Figure 9b still occur, but broaden to larger ranges of the moduli.

As the strain amplitude $\gamma_{0}$ of the sinusoidal deformation increases, both of the samples experience macroscopic yielding, marked by the crossover and following strain-thinning of the two viscoelastic moduli, G${}^{\prime }$ and G${}^{\prime \prime }$, as reported in Figure 7a. However, the evolution followed by the two samples from the peak in loss modulus to the final power law behaviour is different.

**Figure 11:**
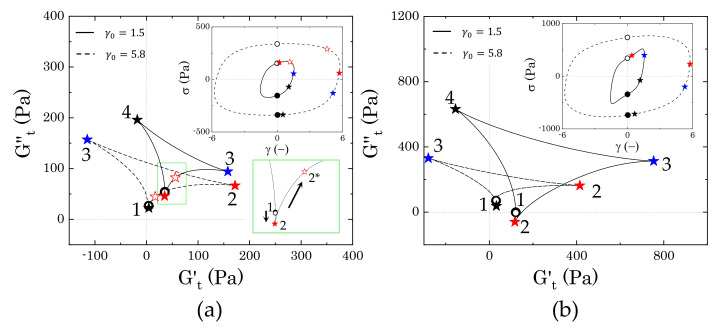
(**a**) Cole-Cole plot obtained at $\gamma_{0}=1.5$ (solid line) and 5.8 (dashed line) (shear-thinning region of the loss modulus) for sample M0 at 2% wt of Carbopol. (**b**) Cole–Cole plot obtained at $\gamma_{0}=1.5$ (solid line) and 5.8 (dashed line) (shear-thinning region of the loss modulus) for sample M2 at 2% wt of Carbopol. The insets show the corresponding elastic Lissajous–Bowditch plot (i.e., stress vs. strain) for reference. The green inset in panel a) zooms closer to the initial steps of the trajectory found for $\gamma_{0}=1.5$.

In the absence of mannitol, the instantaneous trajectories gradually enlarge and evolve from the one reported at the peak of G${}^{\prime \prime }$, where the yielding process begins. In Figure 11a we report two examples, for $\gamma_{0}=1.5$, which is just after the peak of G${}^{\prime \prime }$, and $\gamma_{0}=5.8$, which marks the beginning of the strain-thinning regime of both moduli, thus indicating a complete flow of the material. As larger portions of the material are disrupted, the sequence of intracycle processes experienced by the sample changes. Between the peak of G${}^{\prime \prime }$ and the beginning of the complete flow of the material, the sample shows a nonlinear response that is similar to what is reported in Figure 11a for $\gamma_{0}=1.5$. As the strain increases from an initial zero value, the stress increases weakly with the strain applied, until reaching its maximum, as shown in the Lissajous–Bowditch plot in Figure 11a. In this range of strains, first the instantaneous viscous contribution slightly decreases, reaching its minimum (from point 1 to point 2) and then increases from point 2 to point 2*, as the strain approaches its reversal point. Conversely, $G_{t}^{\prime }$ remains constant in the initial step and then slightly increases up to point 2*. This behaviour indicates a quasi viscoplastic deformation of the material, which, in a packed dispersion of microgels, can be associated with the initial softening and deformation of the particle cages. Afterwards, as the strain reverses, the cages recover their structure, and the instantaneous elastic modulus reaches its maximum (point 3). For a further decrease of the strain, the cages start to deform in the opposite direction, causing an increase in viscous dissipation, which is accompanied by a gradual reduction of the instantaneous elastic contribution, until this reaches a negative value (point 4). A negative value of $G_{t}^{\prime }$ means that, for an increase of strain, the stress reduces (we recall that $G_{t}^{\prime }\equiv \partial_{\gamma }\sigma$ and $G_{t}^{\prime \prime }\equiv \partial_{\dot{\gamma }/\omega }\sigma$); thus, a transition from a positive to a negative $G_{t}^{\prime }$ reflects the transition from a condition, where the strain is partially stored elastically to one where it is completely dissipated viscously [9,31,32]. This indicates that, at point 4, the structure is completely under flow. As the strain approaches zero, the viscous dissipation reduces and the instantaneous elastic modulus gradually recovers until closing the loop again at point 1. As the strain amplitude increases, the sequence of transitions described above remains similar, but the region where the stress is completely dissipated enlarges, until reaching a complete flow of the sample at $\gamma_{0}=5.8$. At this strain amplitude, the instantaneous elastic modulus is negative in the entire range from point 3 to point 1, thus indicating purely viscous disruption of the structure, hence flow.

In the presence of mannitol, the behaviour in the intermediate region of strain amplitudes appears to be qualitatively similar to that observed in the absence of mannitol, yet showing a different evolution of the dimension of the cycles, as can be observed by comparing the evolution of the two trajectories reported in Figure 11a,b. Moving from the condition reported in Figure 10b, the instantaneous trajectories preserve the same features, but increase with the strain amplitude applied, gradually enclosing a larger area, until reaching the maximum area at $\gamma_{0}=1.5$, which corresponds to the small local peak of the storage modulus that is indicated in Figure 7a. The increase of the areas of the trajectories indicate that the internal microstructure progressively experiences broader intracycle deformations as the strain amplitude applied increases, although without showing a complete disruption. At the local peak of G${}^{\prime }$ (i.e., $\gamma_{0}=1.5$), the internal structure has reached its deformation limit. At this strain amplitude, the material first experiences a reduction of the instantaneous viscous modulus to negative values (from point 1 to point 2), which indicates an initial elastic accumulation of the strain that is associated with stretching of the microstructure, followed by a recovery with an increase of both $G_{t}^{\prime }$ and $G_{t}^{\prime \prime }$ (from point 2 to point 3). As the strain reverses, the elastic energy that is accumulated in the previous step is viscously dissipated, indicating a disruption of the internal microstructure, which continues until reaching a purely viscous regime ($G_{t}^{\prime }<0$) (from point 3 to point 4). As the strain applied approaches zero, the viscous dissipation reduces and the structure is partially recovered, as indicated by the increase of the instantaneous elastic modulus, which transitions back to positive values. At increasing strain amplitudes, the instantaneous cycles start to reduce in dimension and assume the same characteristics as those observed in the absence of mannitol. This evolution suggests that, while, in the absence of crystals, the yielding behaviour reflects the disruption/deformation of the particle cages formed by the particles at close contact, the presence of crystals introduces large heterogeneities in the sample, which disrupt the cages. This structure experiences a more complex yielding process, where the initial microstructure that is formed by crystals and particles progressively deforms, stretches, and then completely disrupts in smaller units as the strain amplitude is gradually increased.

#### 3.3.3. Evolution of the Instantaneous Moduli for $c<c_{c}$

**Figure 12:**
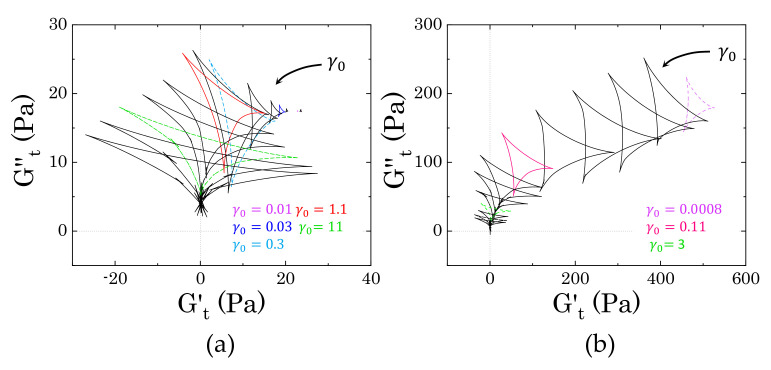
Evolution of the Cole–Cole plots at increasing strain amplitudes for two samples at 0.9% wt of Carbopol (**a**) in the absence of mannitol (sample M0) and (**b**) with mannitol in the presence of crystals (sample M2). All of the deltoids corresponding to the most relevant transitions observed in Figure 7b are marked with a specific colour. For panel (**a**), dashed purple line: first point deviating from the linear viscoelastic plateau; blue solid line: peak of G${}^{\prime \prime }$; cyan dashed line: local minimum of G${}^{\prime \prime }$; red solid line: second peak of G${}^{\prime \prime }$; green dashed line: beginning of final strain-thinning behaviour. For panel (**b**), dashed purple line: first point deviating from the linear viscoelastic plateau; magenta solid line: end of the first decay of G${}^{\prime \prime }$, green dashed line: beginning of final strain-thinning behaviour.

**Figure 13:**
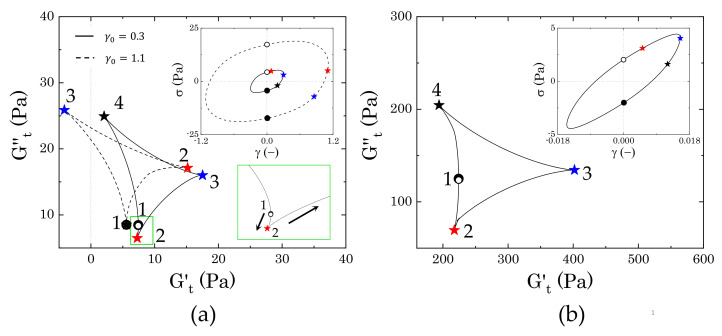
(**a**) Cole-Cole plot obtained at $\gamma_{0}=0.3$ (solid line) and 1.1 (dashed line) (local minimum of G${}^{\prime \prime }$ and second peak of G${}^{\prime \prime }$, respectively) for sample M0 at 0.9% wt of Carbopol; (**b**) Cole–Cole plot obtained at $\gamma_{0}=0.015$ (first region of decay of G${}^{\prime \prime }$) for sample M2 at 0.9% wt of Carbopol. The insets show the corresponding elastic Lissajous–Bowditch plot (i.e., stress vs. strain) for reference.

Below the densely packed regime, the mobility of the particles increases, and the yielding process changes accordingly. Figure 12 reports the evolution of the instantaneous moduli with strain amplitude for a Carbopol mass fraction that is equal to 0.9% wt. As in the previous case, sample M0 clearly shows a narrow extension of the intracycle transitions compared to sample M2, but, in this case, the evolution of the areas of the cycle follows a non-monotonous trend. By comparing Figure 8a with Figure 12a, we notice that the cycles appear to be very similar in the region of strain amplitudes before and after the two peaks of G${}^{\prime \prime }$, thus indicating that the main “cage” structure is preserved, but, thanks to the lower particle densities, the sequence of physical processes associated with particles escape changes in the intermediate range of strain amplitudes, i.e., in the region where the yielding process develops. This is highlighted by the two cycles corresponding to (i) the local minimum of G${}^{\prime \prime }$ following the first small peak ($\gamma_{0}=0.3$) and (ii) the second peak of G${}^{\prime \prime }$ ($\gamma_{0}=1.1$) (Figure 13a). Starting from $\gamma_{0}=0.3$, as the strain increases from zero (point 1), the structure first relaxes, showing a small reduction of both the elastic and viscous instantaneous moduli (point 2), and then gradually recovers, reaching a peak of $G_{t}^{\prime }$ as the strain reverses (point 3). Following the strain reversal, as the shear rate increases, particle bonds break, which causes an increase of the viscous contribution for dissipation and a decrease of the instantaneous elastic modulus, which reaches its minimum as the maximum dissipation is approached (point 4). Finally, as the strain approaches zero, the structure recovers part of its connectivity, causing a gradual increase of $G_{t}^{\prime }$ with a decrease of the viscous dissipation. The intracycle transitions observed at this point indicate the presence of some interparticle bonding, which, at intermediate strain amplitudes, can stretch, causing a temporary alignment of the structure and, therefore, an initial relaxation of the structure.

As the strain amplitude increases, the viscoelastic moduli show a second increase, which culminates in a second peak of the loss modulus accompanied by a small peak of the storage one. At this strain amplitude, the instantaneous cycles resemble those that were observed at higher Carbopol concentrations for a similar strain amplitude: the structure first hardens, with $G_{t}^{\prime }$ reaching its maximum slightly after the strain reversal point, and then disrupts; this results in a gradual increase of the instantaneous viscous modulus and a transition to negative values of the instantaneous elastic modulus. Again, as the strain approaches zero, the structure is gradually recovered and $G_{t}^{\prime \prime }$ reduces to its minimum, closing the cycle. In this case, the rheological transitions indicate a collective structuring and restructuring of the sample, which can be associated with the formation and disruption of the same microstructural units in the entire sample.

In the presence of crystals, the stress response presents nonlinearities from the smallest strain applied, thus indicating that, at this Carbopol concentration, the sample structure is even sensitive to extremely small deformations. The evolution of the instantaneous moduli, as reported in Figure 12b, shows broader rheological transitions at low amplitude strains (corresponding to the first decay of the loss modulus observed in Figure 7b), followed by a reduction of the cycles areas as the material starts to yield. Note that the two plots in Figure 12 have different scales of the coordinates, so it would not be correct to compare the final dimension of the areas in the flow regime. The significant decrease of the areas observed in Figure 12b does not necessarily imply that the final dimension of the microstructural units obtained under flow is smaller than that observed in the absence of mannitol, but it suggests that, in the presence of crystals, a hierarchical structure forms and the material experiences a gradual disruption of its network until reaching smaller units when completely yielded [31].

In the range of deformations corresponding to the first decay of the viscoelastic moduli, where the storage modulus is still higher than the viscous one, the instantaneous trajectories present the same features as the sample cycle that is shown in Figure 13b ($\gamma_{0}=0.015$). As the strain increases, the instantaneous viscous modulus decreases and the elastic one decreases mildly, which indicates an initial strain-induced deformation of the structure that aligns in the direction of the flow. This first transition is followed by a stretching of the structure, resulting in an increase of $G_{t}^{\prime }$ to its maximum that terminates just after the point of strain reversal (point 3). As the strain reduces in the opposite direction, the accumulated stretch is released viscously, generating a reduction of the instantaneous elastic modulus to its minimum and an increase of the viscous one to its maximum (point 4). For a further decrease of the strain, the initial structure recovers, generating a small increase of $G_{t}^{\prime }$ in the early stages, and then keeping a constant elastic contribution as the viscous instantaneous modulus quickly decreases to the initial point. A similar sequence of transitions is observed up to the crossover between G${}^{\prime }$ and G${}^{\prime \prime }$, where the yielding process begins. From that point onward, the cycles reduce in size and show the positive to negative transition of the elastic instantaneous modulus, signalling macroscopic yielding of the structure. Note that, as compared to sample M0, in the presence of crystals the sample yields completely at a lower strain amplitude (dashed green line cycles in Figure 12). This suggests that, even if the crystals generate a more interconnected microstructure causing an increase of the storage modulus at infinitesimal strains, the structure is more fragile, thus allowing for the complete fluidization of the sample at lower strain amplitudes.

## 4. Discussion and Conclusions

In this work, we investigated the effect of the addition of D-mannitol, a common additive in pharmaceutical and personal care product formulations, on the rheological behaviour of non-aqueous Carbopol dispersions while using traditional and time-resolved rheological analysis. When dispersed in PEG400/glycerol solutions, mannitol is metastable; this is revealed by crystals forming after a complete dissolution had been achieved. In light of this, the rheological analysis was performed on two types of samples: (i) regenerated samples (i.e., samples where mannitol is completely dissolved in solution) and (ii) aged samples (i.e., samples presenting the crystalline phase).

In the first case, when completely dissolved in solution, mannitol did not alter the rheological behaviour of the Carbopol dispersions, both in the linear and nonlinear regimes of deformation, as reflected in the superposition of the divergence of the relative viscosity (Figure 4) and the transition of the linear viscoelastic properties as the Carbopol concentration gradually increases (Figure 5). This highlights that the chemical similarity of the additive with the molecules of the surrounding solvent allows for preserving the swollen dimension of the Carbopol particles at equilibrium and, concurrently, the interparticle interactions, which mainly remain governed by the steric interaction between the microgel particles [25,50,53].

On the other hand, the aged solutions presented a shift of the linear viscoelastic properties at higher values, yet without significantly altering the general trends of the loss and storage moduli with the frequency applied. The deviation from the reference sample was modest for the samples containing the lower content of mannitol (sample M1, containing 1.46% wt of mannitol), but was more pronounced for the higher mannitol concentration (sample M2). In both cases, the difference became more evident as the Carbopol concentration was reduced. In particular, for sample M2, we observed a clear shift from a viscoelastic response mainly controlled by the rearrangements of the Carbopol particles to one dominated by the crystals as the Carbopol concentration gradually decreased to zero. These preliminary results indicate that the shape of the crystals in solution influence the change of the rheological properties. In fact, while, for sample M1, the dimension of the crystals is only of a few hundreds of microns and it presents a thin rod-like shape, for sample M2 crystals can reach dimensions of a few millimetres and present a star-like conformation, with denser cores and lateral strands, which can severely interfere with the Carbopol particles in solutions.

To clarify how the crystals interact and alter the microstructure of the samples, we performed large amplitude oscillatory tests and analysed the instantaneous response of the stress to the applied sinusoidal deformation while using the sequence of physical processes (SPP) framework, a method that allows decoupling of the instantaneous elastic contribution from the viscous one, even in the range of large deformations. The analysis was performed for two Carbopol concentrations: (i) one above the closely packed regime, i.e., when the microgel particles are expected to be packed and deformed against their neighbours and (ii) one just below this threshold, when the presence of some interstitial spaces allows higher particle mobility. The evolution of the intracycle transitions that the samples experienced at increasing strain amplitudes revealed clear differences in the yielding process between the reference sample containing only Carbopol (or mannitol non in crystal form) and that containing mannitol crystals.

Above the close packing concentration, the yielding of Carbopol dispersions is expected to be controlled by the balance between the elastic restoring force of the soft particles and the viscous dissipation that is related to particle sliding. The SPP analysis confirms this picture: at low strain amplitudes, the elastic restoring force of the packed particles dominates, the material experiencing a very limited range of transitions; as the amplitude is increased, particle movement is facilitated and the range of intracycle transitions broadens. The cages formed by the packed medium experience a cycle of partial softening and recovery, as the sinusoidal strain is applied. This condition usually corresponds to the maximum of the loss modulus that was observed in LAOS tests and indicates the beginning of the yielding process. As the amplitude of the deformation increases further, the deformation of the cages becomes more significant, until reaching complete cage breaking and, thus, flow. Note that, at this high Carbopol concentration, cages are continuously broken and reformed, because particles are in direct contact, hence maintaining a broad range of intracycle transitions.

In the presence of crystals, the simple “cage” picture is altered by the steric hindrance of the crystals. In this case, the sequence of processes up to the complete yielding of the material is characterised by an initial softening and alignment of the internal structure, which then stretches and breaks, viscously dissipating part of the energy accumulated elastically during the stretching. As the strain amplitude is increased, the sequence is repeated with a broader range of movements, until the structure permanently breaks into smaller units as the material macroscopically flows. This is indicated by the reduction in the size of the cycles in the Cole–Cole plots as the strain increases. A similar reduction has been reported for colloidal gel systems, where the decrease of the cycle area is associated with the rupture of the structure to smaller clusters or even single particles [31]. At this specific concentration, the concentration of Carbopol is too high to allow single particles to break all connections with their neighbours; however, the crystals make the sample heterogeneous, possibly generating local denser particle clusters, which, at low strains, can deform and stretch and, at higher amplitudes, break, causing flow.

As the Carbopol concentration reduces below the close packing value, the solvent gradually occupies larger interstitial spaces between the particles, and the yielding process becomes more sensitive to the specific interactions between the dispersed phases. In the absence of mannitol, the yielding transition reflects the interactions between neighbouring Carbopol particles. In this regime of concentrations, the rheological traces obtained before the first deviation from the linear behaviour and after the yielding process remain unchanged compared to the previous case, thus indicating that particle cages still represent the main microstructural unit of the sample. However, during the yielding process, the intracycle transitions reveal strain accumulation (Figure 12a at γ=0.3), typical of bond stretching [31], which suggests some interparticle attractions at close contact. This observation might be related to the interactions between the external dangling chains of the Carbopol particles, which can interact at close contact, inducing local bridging between particles and forming large disordered particle aggregates.

In the presence of crystals, the yielding process changes noticeably from the previous case. The SPP analysis shows a clear gradual de-structuring of a networked structure, which confers higher elasticity at infinitesimal deformations (as seen in the higher values of G${}^{\prime }$ in Figure 7b), but fails even at small strains, inducing the complete yielding of the system at strain amplitudes an order of magnitude lower than those observed at the same Carbopol concentrations in the absence of mannitol, as seen from the evolution of the Cole–Cole plots in Figure 12b. This behaviour suggests that the initial network is weak and probably induced by direct crystal contacts. To verify this, we performed combined fluorescence and brightfield microscopy imaging on a sample with solvent composition M2 containing 0.7% wt of Carbopol. The sample was prepared following the same procedure that is described in Section 2.2, but using Carbopol particles chemically labelled with rhodamine 123. In this way, crystals could be visualized in bright field mode, thanks to the phase contrast, while Carbopol particles, completely transparent in bright field, were detected in fluorescence mode. A few drops of the fresh dispersion were placed on a glass bottom dish (X20, Fischer Scientific, Leicestershire, UK), sealed, and left over three weeks to allow crystals to form. The same procedure was repeated for the same Carbopol concentration, but in the absence of mannitol, to compare the two main conditions that were investigated through the SPP analysis. The images were taken with an inverted fluorescence microscope (Axio Observer 5, Carl Zeiss Ltd., Cambridge, UK) equipped with a dry phase contrast lens (5x/0.13) and an Axio Cam 506 monochrome camera. The samples were first illuminated in fluorescence mode (LED fluorescence illumination with excitation length of 480 nm) to observe the Carbopol structure and then switched to brightfield mode to capture the crystals. Between the two stages, the sample position and focus remained unchanged.

**Figure 14:**
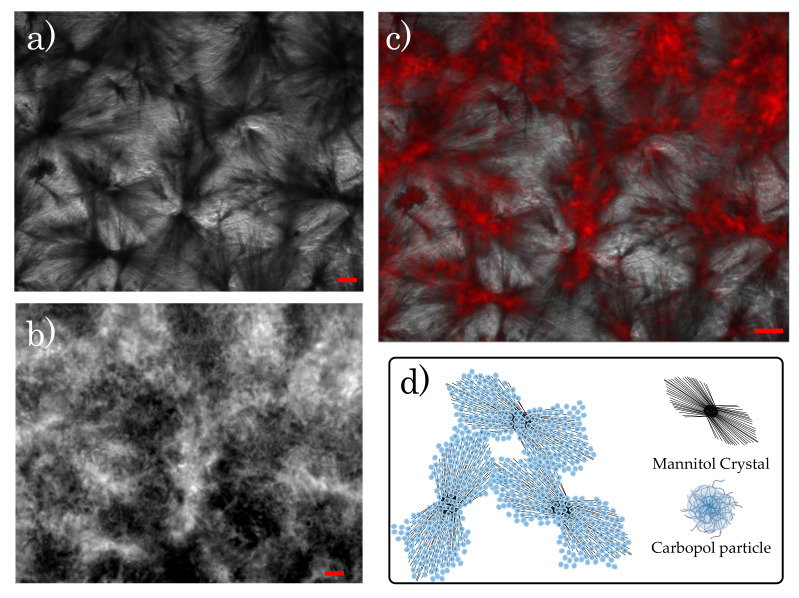
(**a**) Brightfield image of sample M2 at 0.7% wt of Carbopol. Mannitol crystals are the dark areas in the image; (**b**) corresponding fluorescence image of the same sample. In this case, the brighter areas are Carbopol molecules; (**c**) superposition of the two images in panel (**a**,**b**). The red colour of the Carbopol phase was artificially obtained to better highlight the superposition between Carbopol clusters and crystals; and, (**d**) cartoon of the structure observed. The scale bar, reported in red in panels (**a**–**c**) represents a dimension of 150 μm.

**Figure 15:**
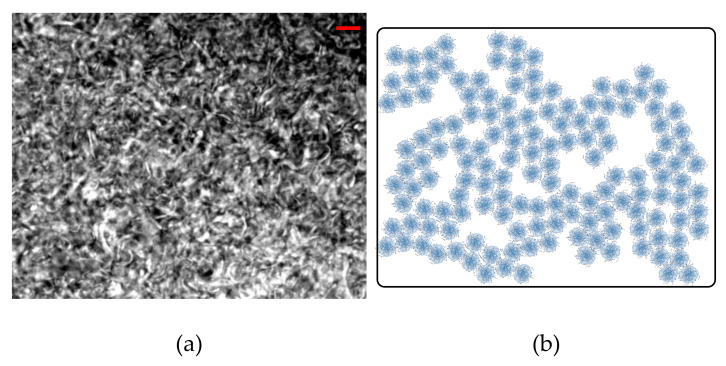
(**a**) Fluorescence image of sample M0 at 0.7% wt of Carbopol (scale bar, reported in red, of 150 μm); (**b**) cartoon of the structure observed in the absence of crystals at the same concentration. Note that a similar structure is observed for fresh or regenerated samples containing mannitol (i.e., in the absence of crystals).

Figure 14 reports the images that were obtained with the two illuminations in the presence of crystals. Note that, in the brightfield images, the crystals are the dark phase because the camera captures the transmitted light, and the bright areas are solvent, while, in fluorescence mode, the camera reads the light emitted by the dye (reflected light), thus showing the Carbopol particles in bright. Even though the resolution of the instrument did not allow visualizing single particles, the general microstructure of the sample was distinguishable. Carbopol molecules appeared to agglomerate close to the crystals. This is better highlighted in Figure 14c, where the two images are superimposed, showing a good match between the areas occupied by the crystals and the brighter regions covered by Carbopol agglomerates. In contrast to the clear agglomerates that were observed in Figure 14b, in the absence of crystals, Carbopol molecules distributed more evenly within the solution, as seen in Figure 15a, where disorderly interconnected bright areas are surrounded by dark pools of solvent. Similar images have already been reported in the literature for aqueous Carbopol gels observed through confocal microscopy with and without mannitol addition [23], showing a larger agglomeration of the Carbopol particles in the presence of mannitol, even though a conclusive explanation for the structure observed was not provided.

By combining the results that were obtained through the rheological analysis and the fluorescence microscopy, we can conclude that, when mannitol is perfectly dissolved or absent, Carbopol particles randomly disperse in solution, without any characteristic microstructural units (Figure 15b), thus showing a rheological behaviour that is consistent with that of soft glassy particle dispersions, where the yielding process is mainly controlled by the deformation and rupture of particle cages. On the other hand, the presence of crystals generates a hierarchical structure, comprising a smaller dispersed phase (Carbopol) that agglomerates around a bigger dispersed phase (crystals) (Figure 14d). Hence, as the concentration of the smaller phase decreases, the relevance of the bigger phase in maintaining the integrity of the microstructure gradually increases and, therefore, from a rheological viewpoint, the local dynamics of the bigger phase gradually control the onset of the yielding process.

However, the molecular origin of the agglomeration process is unclear and further investigation is required to understand the molecular interactions in place. Possibly, the agglomeration process could be related to the adsorption of Carbopol particles on the surface of the crystals by hydrogen bonding, which would be facilitated by the high density of hydroxyl groups that are present on the surface of the mannitol crystals. Further investigation on the nature of the crystalline phase observed and the role of the Carbopol molecules on the growth and final geometrical configuration of the crystals could confirm this hypothesis and clarify the mechanism behind the agglomeration process.

## Supplementary Materials

The following are available online at https://www.mdpi.com/article/10.3390/ma14071782/s1, Table S1: Conversion table for samples compositions.
