# Supplementary material for: Effect of D-Mannitol on the Microstructure and Rheology of Non-Aqueous Carbopol Microgels
Source: Materials (Basel). 2021 Apr 4;14(7):1782. doi: 10.3390/ma14071782 (PMC8038445; doi:10.3390/ma14071782)
Supplement: Supplementary file 1 [file materials-14-01782-s001.pdf]

materials

MDPI

supplementary

Effect of D-Mannitol on the Microstructure and Rheology of Non-Aqueous Carbopol Microgels

Simona Migliozzi, Panagiota Angeli *, and Luca Mazzei *

Department of Chemical Engineering, University College London, Torrington Place, London WC1E 7JE, UK; simona.migliozzi.16@ucl.ac.uk
* Correspondence: p.angeli@ucl.ac.uk (P.A.); l.mazzei@ucl.ac.uk (L.M.)

Citation: Migliozzi, S.; Angeli, P.; Mazzei, L. Effect of D-Mannitol on the Microstructure and Rheology of Non-Aqueous Carbopol Microgels. *Materials* **2021**, *14*, 1782. https://doi.org/10.3390/ma14071782

Academic editor: Yannis Dimakopoulos

Received: 17 March 2021
Accepted: 1 April 2021
Published: 4 April 2021

Publisher's Note: MDPI stays neutral with regard to jurisdictional claims in published maps and institutional affiliations.

Copyright: © 2021 by the authors. Submitted for possible open access publication under the terms and conditions of the Creative Commons Attribution (CC BY) license (http://creativecommons.org/licenses/by/4.0/).

Table 1. Exact compositions of all samples used in terms of % wt (w_c) and mass concentration c (g/mL). The first column reports the nominal percentage weights for each sample.

Nominal %wt	M0		M1		M2	
	w_c (%)	*c* (g/mL)	w_c (%)	*c* (g/mL)	w_c (%)	*c* (g/mL)
0.05	0.052	6.26×10^{-4}	0.055	6.7×10^{-4}	0.051	6.25×10^{-4}
0.1	0.1	1.21×10^{-3}	0.12	1.15×10^{-3}	0.11	1.38×10^{-3}
0.15	0.15	1.8×10^{-3}	0.15	1.53×10^{-3}	0.16	1.84×10^{-3}
0.2	0.2	2.43×10^{-3}	0.2	2.74×10^{-3}	0.21	2.56×10^{-3}
0.3	0.3	3.65×10^{-3}	0.3	3.7×10^{-3}	0.3	3.66×10^{-3}
0.35	0.35	4.25×10^{-3}	0.346	4.2×10^{-3}	0.356	4.32×10^{-3}
0.4	0.4	4.86×10^{-3}	0.4	4.84×10^{-3}	0.396	4.81×10^{-3}
0.5	0.5	6.08×10^{-3}	0.5	6.09×10^{-3}	0.506	6.15×10^{-3}
0.6	-	-	0.598	7.26×10^{-3}	0.601	7.29×10^{-3}
0.7	0.7	8.49×10^{-3}	0.694	8.42×10^{-3}	0.73	8.86×10^{-3}
0.8	-	-	0.796	9.66×10^{-3}	0.826	1×10^{-2}
0.9	0.902	1.09×10^{-2}	0.89	1.08×10^{-2}	0.908	1.1×10^{-2}
1	1	1.21×10^{-2}	0.98	1.19×10^{-2}	1	1.21×10^{-2}
1.5	1.5	1.82×10^{-2}	1.49	1.8×10^{-2}	1.5	1.82×10^{-2}
2	1.63	1.98×10^{-2}	1.99	2.42×10^{-2}	2	2.42×10^{-2}
3	3.01	3.65×10^{-2}	2.99	3.62×10^{-2}	2.86	3.47×10^{-2}
